# Amyloidosis Associated Kidney Failure with Gross Hypermetabolic Intra-abdominal Mass

**DOI:** 10.4274/mirt.galenos.2019.92485

**Published:** 2020-02-17

**Authors:** Zehra Pınar Koç, Pınar Pelin Özcan, Kenan Turgutalp, Kaan Esen, Tuğba Kara

**Affiliations:** 1 Mersin University Faculty of Medicine, Department of Nuclear Medicine, Mersin, Turkey; 2 Mersin University Faculty of Medicine, Department of Nephrology, Mersin, Turkey; 3 Mersin University Faculty of Medicine, Department of Radiology, Mersin, Turkey; 4 Mersin University Faculty of Medicine, Department of Pathology, Mersin, Turkey

**Keywords:** Amyloidosis, abdominal mass, 18F-FDG positron emission tomography/computed tomography

## Abstract

A 23-year-old male patient who presented with impaired kidney function tests attended to hospital for hemodialysis and underwent 18F-FDG positron emission tomography/computed tomography (PET/CT) examination for the metabolic characterization of the intra-abdominal mass which was found in the ultrasonography. ^18^F-FDG PET/CT revealed a mass lesion adjacent to the liver which was hypermetabolic and the pathology of the lesion was determined as amyloidosis. To the best of our knowledge, the case with ^18^F-FDG PET/CT images of a huge amyloid mass is the first in the literature.

## Abstract (tr)

Yirmi üç yaşında erkek hasta böbrek yetmezliği ile diyaliz için hastanemize yönlendirilmiştir. Hastanın ultrasonografisinde tesadüfen saptanan intra-abdominal kitle lezyonunun metabolik karakterizasyonu için yapılan ^18^F-FDG pozitron emisyon tomografi/bilgisayarlı tomografi (PET/BT) çalışmasında hipermetabolik kitle izlenmiş olup kitlenin biyopsisi amiloidozis ile uyumlu olarak bulunmuştur. Bilgilerimize göre, bu olgu ile, dev amiloid kitlesi ile başvuran bir hastanın ^18^F-FDG PET/BT görüntüleri literatürde ilk kez sunulmaktadır.

## Figures and Tables

**Figure 1:**
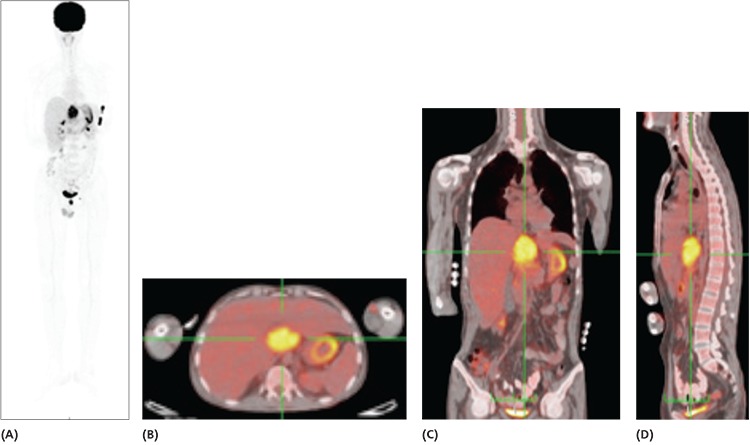
**A)** ^18^F-FDG positron emission tomography/computerized tomography (PET/CT) maximum intensity projection image showing hypermetabolic intra-abdominal mass standardized uptake value (SUV_max_: 5) adjacent to the liver, spleen and stomach and an additional hypermetabolic left servical lymph node (SUV_max_: 3.6). **B, C, D)** Cross sectional images of the same intraabdominal mass in transaxial, coronal and sagittal projections. A 23-year-old male patient presented with sudden onset acute kidney failure and the patient was referred for hemodialysis. The abdominal ultrasonography revealed intra-abdominal mass adjacent to the liver and the patient was referred to the Nuclear Medicine Department with pre-diagnosis of plasmocytoma. ^18^F-FDG PET/CT imaging showed hypermetabolic intra-abdominal mass and a mildly hypermetabolic left cervical lymph node which was thought to be scondary to an infection. The hypermetabolic abdominal mass was diagnosed as amyloid deposition via true-cut biopsy.

**Figure 2:**
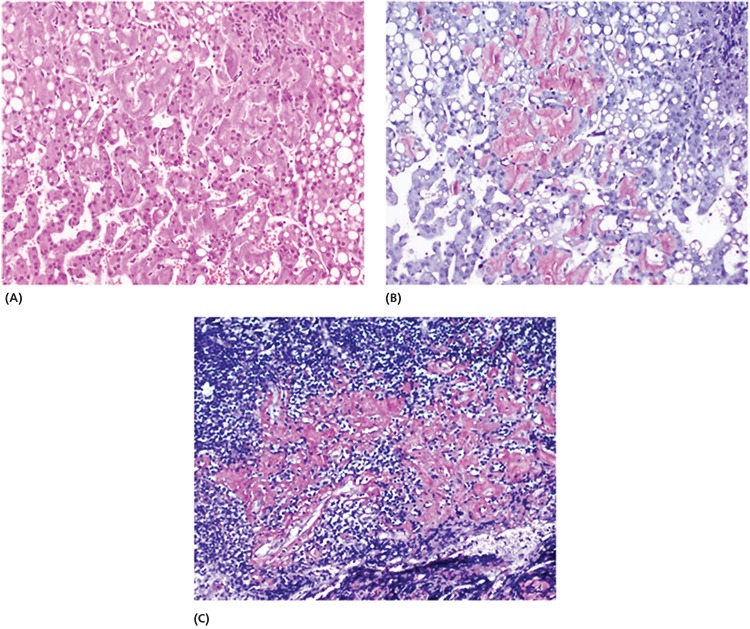
Abdominal lymph node biopsy and wedge resection from the liver parenchyma and adjecent lymph node were performed. The pathological result of the mass was found to be an amyloid deposition. Additionally, amyloid deposits were also found in the hepatocytes. **A)** Acellular, amorphous, eosinophilic amyloid deposits in hepatocyte cytoplasm (hematoxylin and eosin, x200), **B)** amyloid was stained brown to orange with Kongo Red dye in the hepatocyte cytoplasm (Kongo Red, x200), **C)** amyloid deposits in the lymph node parenchyma (Kongo Red, x200). In microscopic examination, acellular, amorphous, eosinophilic deposits were detected in the lymph node parenchyma and also in the hepatocyte cytoplasm and disse space in the liver parenchyma. Additionally depositions were shown in the vascular walls indicating systemic amyloidosis. The deposits were stained brown to orange with Kongo Red dye and also showed birefringence under polarized light, which was compatible with amyloidosis. To specify the type of amyloid proteins immunohistochemically, AA amyloid dye was applied to the biopsy materials and positive staining was observed. Primary amyloidosis presented with hepatic involvement is a rare disease that was previously reported in some cases with multiple myeloma (1). Additionally, there are case reports about ^18^F-FDG accumulation of primary amyloidosis in the lungs (2). A previous case report showed diffuse increased liver ^18^F-FDG uptake (3), however the series comparing systemic and localized amyloidosis indicate that systemic amyloidosis may not accumulate ^18^F-FDG but localized amyloidosis does (4). The lesion in this report was located in close proximity to the liver, spleen and stomach but not in the any of these organs. The probable reason for the kidney impairment was amiloidosis as well. The prediagnosis of multiple myeloma or plasmocytoma was excluded by laboratory analysis. Kidney biopsy was not performed however amyloid deposition in the normal liver parenchyma was shown by pathology results demonstrating systemic amlioidosis. However the reason of this amiloidosis could not be determined. The patient also has some ^18^F-FDG uptake in gastric region which was explained by gastritis. In the literature, this is the first case report with systemic and localized amyloidosis presented with kidney failure and gross abdominal mass showing high ^18^F-FDG uptake.
